# Silicon nanoparticles enhance maize yield and water productivity via regulating photosynthesis and canopy structure under mild regulated deficit irrigation

**DOI:** 10.3389/fpls.2025.1691443

**Published:** 2026-01-06

**Authors:** Xukai Liang, Qi Liao, Panpan Guo, Ziyi Yang, Shaozhong Kang, Taisheng Du, Ling Tong, Risheng Ding

**Affiliations:** 1 Center for Agricultural Water Research in China, China Agricultural University, Beijing, China; 2 State Key Laboratory of Efficient Utilization of Agricultural Water Resources, Beijing, China; 3 National Field Scientific Observation and Research Station on Efficient Water Use of Oasis Agriculture, Wuwei, Gansu, China

**Keywords:** silicon nanoparticles, regulated deficit irrigation, leaf inclination angle, canopy intercepted net radiation, gas exchange

## Abstract

**Introduction:**

Silicon nanoparticles (SiNPs) effectively mitigate drought stress in crops, yet their physiological mechanisms in maize remain unclear.

**Methods:**

This study conducted field experiments in the arid region of northwest China, setting up three maize genotypes (Zhengdan 958, Zhongdan 2, and MC670), two irrigation methods (full irrigation, FI, and regulated deficit irrigation, RDI), and two exogenous treatments (water as control, and SiNPs application).

**Results:**

The RDI increased stomatal density (*SD*), intrinsic water use efficiency (iWUE), and water productivity (WP), albeit with a slight reduction in yield. However, the application of SiNPs increased the yield and WP across all three genotypes under both FI and RDI. Additionally, SiNPs notably enhanced SPAD values, stomatal conductance (*g*_s_), net photosynthesis rate (*A*), leaf area index (LAI), and the fraction of photosynthetically active radiation (*f*_PAR_), while reducing the leaf inclination angle (LIA) at the middle ear position. Further analysis revealed the following mechanisms: (1) an increase in SPAD and *g_s_* enhanced *A*; (2) enhanced LAI and reduced LIA at the ear-bearing canopy layers significantly improved *f*_PAR_; and (3) the combined increase in *A* and *f*_PAR_ synergistically contributed to increased maize yield. The improvements in WP were more strongly correlated with yield gains than with changes in evapotranspiration.

**Discussion:**

The findings demonstrate that SiNPs improve maize productivity and water use efficiency under both full and deficit irrigation by coordinately enhancing photosynthetic performance and optimizing canopy light interception. The results provide physiological insights into how SiNPs alleviate drought-related limitations in maize. These findings offer important theoretical insights and a practical strategy for employing SiNPs as a sustainable crop enhancer under water-limited conditions.

## 1 Introduction

Food security is an indispensable cornerstone of national security, and the continuous growth of the population and climate change have brought unprecedented challenges to the utilization of cropland and water resources (Ray et al., 2012; Myers et al., 2017; Yang et al., 2025). It is estimated that global food production needs to double by 2050 to meet the expected demand driven by population growth, dietary changes, and increased biofuel consumption (Ray et al., 2013). This goal places higher demands on the production of staple crops, especially maize, which is the world’s largest grain crop. Data indicate that in 2023, the global maize yield was 5962.3 kg ha^-^¹, with a planting area of 208.2 million ha (FAOSTAT., 2025). Drought is one of the major abiotic stresses limiting crop yield, as it suppresses photosynthesis and induces early senescence, ultimately resulting in a significant reduction in crop yield (Morales et al., 2020; Li et al., 2023). Therefore, exploring effective drought-resistant strategies to ensure stable crop production and increase efficiency has become an urgent issue to be addressed in current agricultural research.

The Green Revolution significantly increased crop yields by improving the harvest index (HI). However, as HI approaches its theoretical limit, optimizing photosynthetic efficiency has become a critical direction for overcoming yield bottlenecks (Long et al., 2006, Long et al., 2015; Ort et al., 2015). Photosynthesis is not only the key process for converting light energy into chemical energy but also the core determinant of crop yield and water productivity (Karami et al., 2025; Feller, 2016). Under drought conditions, crops exhibit hierarchical responses in their photosynthetic systems. At leaves level, stomatal and mesophyll limitations reduce CO_2_ availability, decreasing photosynthetic rates under the early drought. As drought intensifies, oxidative stress further suppresses photosynthesis (Loreto and Centritto, 2008; Ors et al., 2021). To cope with this dual stress, crops regulate physiological processes to maximize carbon assimilation and minimize water loss, including adjustments in stomatal anatomy and enhancements in antioxidant defenses (Munjonji and Ayisi, 2021; Sabino et al., 2021). At the canopy scale, crops employ morphological adaptation strategies to optimize photosynthetic performance. For example, maize regulates its leaf area index (LAI), leaf angle, and spatial arrangement to enhance canopy structure. These adjustments significantly improve light distribution within the canopy, increase light interception efficiency, and reduce water transpiration losses (Liu et al., 2022). Among these, leaf area and leaf angle play decisive roles in the interception of photosynthetically active radiation, which directly affects canopy photosynthesis and final yield (Stewart et al., 2003). The optimization of canopy structure by regulating the vertical distribution of plant leaf area and leaf angles has received widespread attention to meet the crop production demand at present studies (Perez et al., 2019; Utsugi et al., 2006; Li et al., 2025). Existing studies often focus on isolated responses at the leaf or canopy scale, while the mechanisms of multilevel synergistic optimization between leaf-scale and canopy-scale responses to enhance light interception remain to be further explored.

The traditional high-yield irrigation theory (full irrigation) advocates providing sufficient water throughout the entire crop growth cycle to pursue maximum yield. However, when water supply exceeds a certain threshold, further increases contribute little to yield (Jiao et al., 2024). To address this issue, the regulated deficit irrigation (RDI) approach has been developed, primarily aimed at reducing water consumption during non-critical growth stages while maintaining stable yields and improving water use efficiency (Pardo et al., 2020). Its robustness has been validated by several studies (Wang et al., 2023; Jiao et al., 2024; Liu et al., 2025). Additionally, our previous research revealed that mild RDI promotes partial stomatal closure through osmotic regulation while maintaining photosynthetic capacity, ultimately improving water use efficiency (Liao et al., 2022). The changes in stomatal density (*SD*) also participate in plant responses to RDI-induced water stress. However, the positive (Bosabalidis and Kofidis, 2002; Fraser et al., 2009; Fu et al., 2013) or negative (Quarrie and Jones, 1977; Xu and Zhou, 2008) effects of RDI on *SD* depend on the species and water stress conditions.

Nanomaterials are gradually emerging as a promising tool in overcoming biotic and abiotic stresses. Among these, Silica Nanoparticles (SiNPs) have attracted widespread attention due to their excellent chemical and optoelectronic properties (Yuvaraj et al., 2023). SiNPs possess mesoporous structures, are easy to synthesize, and exhibit good biocompatibility, low toxicity, and excellent thermal stability (Naidu et al., 2023). As a beneficial trace nutrient for plants, the addition of SiNPs has positive effects on the growth, development, and productivity of various plants (Zulfiqar et al., 2019; Nazim et al., 2024; Mukarram et al., 2022). Research has shown that SiNPs can mitigate the negative effects of drought stress on ion balance, pigment content, and oxidative stress in eggplant plants (Younes et al., 2024), promote root development in *Cunninghamia lanceolata*, and enhance gas exchange (Liu et al., 2023). Additionally, SiNPs can promote growth and photosynthetic performance in lemongrass under salt stress and activate its enzymatic antioxidant system (Mukarram et al., 2023), while also increasing chili yield in saline-alkaline environments (Li et al., 2024). Studies have shown that nanomaterials can regulate leaf growth and angle (Guo et al., 2006; Abd-El-Aty et al., 2024; Liu et al., 2012), but the mechanisms between these changes and maize production remain unclear. Furthermore, current research lacks an in-depth analysis of the interactive mechanisms between water regulation effects and silicon nutrient enhancement when RDI is combined with SiNPs application. Additionally, no systematic research framework has been established to elucidate the physiological and ecological mechanisms of yield formation based on their combined effects.

Irrigation practices are usually integrated with traditional management measures, such as fertilization, to achieve efficient agricultural production. Given the water-saving potential of deficit irrigation and the ability of SiNPs to enhance crop stress resistance, we highlight the close link between crop production and its physiological and ecological processes. Thus, we raise the following questions: (1) Can nano-silicon improve maize yield and water productivity under various irrigation conditions? (2) Is the increase in maize yield and water productivity from nano-silicon related to the optimization of single-leaf photosynthetic rate and canopy structure? (3) Can combining nano-silicon with deficit irrigation lead to stable and efficient crop production? To address these questions, this study conducted field experiments in a typical arid region of Northwest China to analyze the effects and mechanisms of different irrigation methods and exogenous substance treatments on crop yield and water productivity and explore the potential of nano-silicon in agriculture.

## 2 Materials and methods

### 2.1 Experimental site and experimental design

The experiment was conducted in 2024 at the National Field Scientific Observation and Research Station for Efficient Water Use in Oasis Agriculture, located in Wuwei, Gansu Province, China (37°51′N, 102°52′E). The experimental site is characterized by a temperate continental arid climate, with annual sunshine hours exceeding 2830 h. The long-term average precipitation and evaporation are 263 mm and 2000 mm, respectively, and the groundwater depth is below 25 m, posing a high risk of drought for agricultural production.

In this study, three representative maize genotypes widely cultivated in Northwest China since 1977 were selected: Zhongdan 2 (ZD2#), Zhengdan 958 (ZD958), and MC670. Each variety was subjected to two irrigation levels (W, water treatment): full irrigation (FI) and regulated deficit irrigation (RDI), and two exogenous substance treatments (B, biostimulants treatment): H_2_O for control (H_2_O) and silica nanoparticles (SiNPs). Each treatment was replicated three times, with a total of 36 plots. The area of each experimental plot was 24 m² (4 m × 6 m), with a row spacing of 40 cm and plant spacing of 25 cm. The soil type of the experimental site was sandy loam, with a bulk density of 1.53 g cm^-^³ and a field capacity of 0.31 m³ m^-^³ within the 100 cm soil layer. An automatic weather station located 200 m from the experimental site monitored meteorological parameters every 15 minutes. The saturated vapor pressure deficit (VPD, kPa), precipitation (P, mm), and reference crop evapotranspiration (ET_0_, mm) during the entire maize growing season are shown in **Supplementary Figure S1**.

The irrigation amount for FI treatment was determined based on the predicted crop water requirement and further adjusted according to the actual soil water deficit. The predicted crop water requirement was estimated using the dual crop coefficient method recommended by FAO-56, with the selection of the basal crop coefficient referring to Jensen and Allen. Regulated deficit irrigation was applied with a target of 65% and 80% of the irrigation amount applied in the FI treatment during the same period for the late vegetative (V8-VT, eight leaves to tasseling stages) and maturation (R4-R6, dough to physiological maturity stages) growth-stage periods, respectively. Before and after each irrigation, as well as at sowing and harvesting, soil water content (SWC) was measured using a neutron soil moisture meter at 20 cm intervals within a soil depth of 100 cm. The changes in SWC under different treatments during the maize growth period are shown in **Supplementary Figure S2**. Irrigation was conducted using subsurface drip irrigation, and nitrogen fertilizer was applied at a rate of 250 kg ha^-^¹ throughout the growth period. The irrigation schedule is presented in **Table 1**.

**Table 1:** Irrigation amount (mm) for different treatments of the three maize genotypes. Treatments: control, FI_H_2_O; regulated deficit irrigation, RDI_H_2_O; nanosilicon exogenous additive, FI_SiNPs; combined regulated deficit irrigation and nanosilicon exogenous additive, RDI_SiNPs.

Maize genotypes	treatmentDate (mm/dd)	06/08	06/25	07/10	07/25	08/12	08/31	Entire growth period
ZD958	FI_H_2_O	34.86	64.49	55.36	69.75	37.77	30.98	293.20
	RDI_ H_2_O	20.91	43.66	66.43	69.75	30.22	24.78	255.75
	FI_SiNPs	34.86	64.49	55.36	69.75	37.77	30.98	293.20
	RDI_SiNPs	20.91	43.66	59.49	75.06	30.22	24.78	254.12
ZD2#	FI_ H_2_O	34.86	64.49	55.36	69.75	37.77	30.98	293.20
	RDI_ H_2_O	20.91	43.66	66.43	69.75	30.22	24.78	255.75
	FI_SiNPs	34.86	64.49	55.36	69.75	37.77	30.98	293.20
	RDI_SiNPs	20.91	43.66	66.43	75.06	30.22	24.78	261.06
MC670	FI_ H_2_O	34.86	64.49	80.16	85.67	37.77	30.98	333.92
	RDI_ H_2_O	20.91	43.66	96.19	85.67	30.22	24.78	301.43
	FI_SiNPs	34.86	64.49	80.16	85.67	37.77	30.98	333.92
	RDI_SiNPs	20.91	43.66	89.24	85.67	30.22	24.78	294.49

SiNPs (approximately 20 nm in diameter) were purchased from Shanghai Pantian Powder Material Co., Ltd. On the day before sowing, a 40 mg L^-^¹ SiNPs solution was prepared and sonicated for 30 minutes using an ultrasonic machine. Uniform and fully developed maize seeds were selected and disinfected by soaking in 2% sodium hypochlorite solution for 15 minutes, followed by rinsing three times with distilled water. Subsequently, the seeds were soaked in either 40 mg L^-^¹ SiNPs solution or pure water (H_2_O treatment) for 12 hours in the dark at an ambient temperature of 25 °C. The three maize genotypes were sowing on April 29. After the maize entered the jointing stage, foliar application of exogenous substances was performed in the evening on June 11, June 18, and June 26. The control treatment was sprayed with distilled water, while the SiNPs treatment was sprayed with a 150 mg L^-1^ SiNPs solution. The SiNPs dispersion was ultrasonicated for 30 minutes to ensure nanoparticle monodispersity. During application, using a hand-held sprayer equipped with a fine atomizing nozzle under consistent pressure, the solution was converted into a fine mist and applied through multiple light passes over the leaves. The leaf surface condition was closely monitored to ensure complete wetness without droplet accumulation, with immediate cessation when any tendency for droplet formation was observed. The application dosage was progressively increased as the maize canopy developed. The spraying dosage was about 926 L ha^-1^ on June 11, 1157 L ha^-1^ on June 18, and 1620 L ha^-1^ on June 26.

### 2.2 Measurements of leaf gas exchange and stomatal density

Leaf gas exchange was measured between 9:00 and 12:30 using a portable photosynthesis system (LI-6800, LICOR Biosciences, Lincoln, NE, USA). Measurements were conducted on July 1 to 3 for the seventh leaf from the top, and on July 18 to 20 and August 17 to 19 for the ear leaf. The leaf chamber environment was set as follows: relative humidity of 60%, PPFD of 1800 μmol m^-^² s^-^¹, and reference CO_2_ concentration of 400 μmol mol^-^¹. The measured parameters included stomatal conductance (*g*_s_, mol m^-2^ s^-1^), net photosynthetic rate (*A*, μmol m^-2^ s^-1^), intrinsic water use efficiency (iWUE=*A/g*_s_, μmol mol^-1^). After the measurements, the imprint method was immediately used to collect stomatal anatony. A layer of colorless transparent nail polish was applied to the adaxial and abaxial surfaces of the leaf. Once the nail polish dried, it was carefully peeled off using transparent tape and mounted onto a glass slide. For each sample, three random fields of view were selected and photographed under an optical microscope (CX33, Olympus, Tokyo, Japan). Stomatal density (*SD*, No. mm^-2^) on the adaxial and abaxial surfaces was analyzed using ImageJ software.

### 2.3 Measurements of leaf area index, plant height, biomass, leaf inclination angle, canopy radiation interception rate, and SPAD values

On June 4, June 18, July 15, August 5, September 11, and September 18, two representative maize plants were randomly selected from each plot. Plant height and one-sided green leaf area were measured using a tape measure. The area of a single leaf was calculated as length × width × 0.75, and the total leaf area per plant was the sum of all green leaf areas. The leaf area index (LAI, m^2^ m^-2^) was calculated as the ratio of leaf area to ground area. The aboveground parts of the plants were oven-dried at 85 °C until constant weight, and biomass (kg m^-2^) was calculated based on a unit area.

On July 20, leaf inclination angles were measured using a digital angle meter, including the ear leaf and four leaves above and below the ear leaf. For analysis, the leaf inclination angle (LIA) was determined for the upper, middle, and lower canopy layers, defined relative to the ear leaf. The middle layer consisted of three leaves: the ear leaf and the ones immediately above and below it. The upper and lower layers each comprised the three leaves immediately adjacent to the middle layer, above and below it, respectively. The LIA for each layer was calculated as the average of all leaves within it. On June 23, July 3, July 24, and August 4, the radiation at 0.05 m above the ground surface under the canopy and 0.05 m at the top of the canopy was measured using an ACCUPAR (LP-80, Decagon, USA). The fraction of canopy radiation interception (*f*_PAR_) was calculated as follows:


$$f_{\text{PAR}}=\frac{{\text{PAR}}_{\text{top}}-{\text{PAR}}_{\text{bottom}}}{{\text{PAR}}_{\text{top}}}$$

where PAR_top_ (μmol m^-2^ s^-1^) is the radiation at the top canopy; and PAR_bottom_ (μmol m^-2^ s^-1^) is the radiation at the bottom of the canopy. SPAD values of the ear leaf were measured using a portable chlorophyll meter (SPAD-502 plus; Konica Minolta, Japan) on July 5, August 4, and August 25.

### 2.4 Yield and its components, crop water requirement, and water productivity

Maize was harvested during the black layer formation stage at the base of the grain. Zhongdan 2 (ZD2#) harvested on September 12, and MC670 and Zhengdan 958 (ZD958) harvested on September 19. A total of 30 ears from the two central rows of each plot were selected as yield samples. Three ears were randomly chosen from these, and ear diameter was measured using a caliper with an accuracy of 0.02 mm, while ear length was measured with a ruler with an accuracy of 1 mm. The length of the tip, number of kernel rows, number of kernels per row, and total number of kernels per ear were recorded to evaluate the yield components. The remaining ears were threshed and placed in an 85 °C constant temperature drying oven until reaching a constant weight to measure yield (kg ha^-1^; 14% moisture content) and thousand-kernel weight (*g*; 14% moisture content). Crop evapotranspiration (ET, mm) during the entire growth period was calculated using the water balance method. Water productivity (WP, kg m^-3^) was calculated as the ratio of yield to ET.

### 2.5 Silica nanoparticles

The particle size and morphology of nano-silicon particles (SiNPs) were characterized using a transmission electron microscope (TEM-EDS, JEM F200, Japan). Infrared spectra were collected using a Fourier transform infrared spectrometer (FTIR, Thermo Nicolet IS5). The zeta potential of SiNPs was measured using a nanoparticle size and zeta potential analyzer (Zetasizer Pro Blue). The release of hydroxyl radicals in silica was determined by electron paramagnetic resonance (EPR, 200M, CIQTEK, China) spectroscopy.

### 2.6 Statistical analysis

Analysis of variance was performed using SPSS 26.0 (SPSS, Chicago, Illinois, USA). Graphs were created using Origin 2024 (OriginLab Corporation, Northampton, MA, USA). The response value of each indicator was calculated as (experimental value − control value)/control value, where the control value refers to the FI_H_2_O treatment. The standard deviation was calculated using the error propagation law. All statistical tests were considered significant at *p* < 0.05.

## 3 Results

### 3.1 Characterization of SiNPs

TEM analysis revealed that the nanoparticles exhibited a spherical morphology with diameters of approximately 15–30 nm. Extensive aggregation was observed, indicating strong electrostatic attractions between the biomolecular stabilizers present on the surface of SiNPs (**Supplementary Figures S3a, b**) (Ahmed et al., 2023). FTIR spectral analysis revealed absorption peaks at 3444.19 cm^-^¹ and 1636.93 cm^-^¹, corresponding to the characteristic absorption of O–H bonds (Sarkar et al., 2025), while the peaks at 1104.09 cm^-^¹, 799.75 cm^-^¹, and 469.34 cm^-^¹ were attributed to the characteristic absorption of Si–O–Si bonds (**Supplementary Figure S3c**) (Ale et al., 2023). Zeta potential analysis showed a surface charge of -32.37 mV (**Supplementary Figure S3d**) (Shafqat et al., 2025). EPR analysis detected that SiNPs could catalyze the generation of hydroxyl radicals (·OH) (**Supplementary Figure S3e**).

### 3.2 Effects of different irrigation methods and exogenous substances on yield and its components

**Table 2** presents the effects of different irrigation methods and exogenous substance treatments on the growth period, yield, and its components of the three maize genotypes. Compared to FI treatment, RDI treatment significantly reduced ear diameter, 1000-kernel weight, and yield in both H_2_O and SiNPs treatments. Compared to H_2_O treatment, SiNPs significantly increased 1000-kernel weight and yield under both FI and RDI. Except for the harvest index, there were significant differences in yield and its components among genotypes, with MC670 having the highest yield, followed by ZD958, and lastly by ZD2#. For ZD958, the yield changes compared to FI_H_2_O treatment were -0.99%, 6.74%, and 3.05% for RDI_H_2_O, FI_SiNPs, and RDI_SiNPs treatments, respectively. For ZD2#, the corresponding yield changes were -6.92%, 8.88%, and -0.52%, while MC670 showed yield changes of -19.88%, 2.97%, and -4.67%.

**Table 2:** Effects of different water and exogenous substance treatments on the growth period, yield, and yield components of three maize genotypes.

Genotypes	Treatments	Anthesis DOY	Silking DOY	Anthesis-silking interval	Ear length/cm	Ear diameter/cm	Bare cusp length/cm	Row number per ear	Kernel numbers per row	Kernel number per ear	Thousand kernels weight/g	Yield/(kg·ha^-1^)	Harvest index
ZD958	FI_H_2_O	187.33	186.33	-1.00	16.31	53.73	0.99	15.78	31.64	492.06	334.23	15349.49	0.51
	RDI_H_2_O	187.33	188.33	1.00	16.58	52.00	1.39	15.00	32.75	474.94	320.96	15197.21	0.50
	FI_SiNPs	187.67	186.00	-1.67	16.38	53.92	0.96	15.78	32.42	506.67	353.02	16383.61	0.49
	RDI_SiNPs	186.67	187.00	0.33	16.62	52.53	1.26	15.11	31.89	480.44	345.05	15817.06	0.51
ZD2#	FI_H_2_O	186.00	188.33	2.33	20.26	48.71	1.96	14.00	36.11	493.44	314.02	15013.84	0.43
	RDI_H_2_O	186.00	189.00	3.00	20.38	48.68	2.41	14.37	34.26	444.41	319.01	13974.82	0.52
	FI_SiNPs	186.00	188.33	2.33	20.88	49.53	2.18	13.56	37.28	488.67	338.35	16346.68	0.46
	RDI_SiNPs	186.67	187.00	0.33	20.11	49.23	1.98	14.22	36.56	499.00	328.17	14936.41	0.51
MC670	FI_H_2_O	186.33	186.00	-0.33	18.41	53.28	2.45	18.50	32.90	580.21	310.89	17934.47	0.52
	RDI_H_2_O	186.67	187.33	0.67	18.13	49.28	2.36	18.44	34.22	608.00	279.04	14369.04	0.43
	FI_SiNPs	186.00	186.00	0.00	18.88	51.69	2.42	17.22	34.31	567.17	310.50	18467.78	0.50
	RDI_SiNPs	186.33	186.33	0.00	18.88	51.87	2.13	18.44	34.86	606.83	301.21	17096.07	0.49
Three-way ANOVA	W	ns	*	ns	ns	*	ns	ns	ns	ns	**	***	ns
	B	ns	*	*	ns	ns	ns	ns	ns	ns	***	**	ns
	G	**	***	***	***	***	***	***	***	***	***	***	ns
	W×B	ns	*	ns	ns	ns	ns	ns	ns	ns	ns	ns	ns
	W×G	ns	ns	**	ns	ns	ns	ns	ns	ns	ns	*	ns
	B×G	ns	ns	ns	ns	ns	ns	ns	ns	ns	ns	ns	ns
	W×B×G	ns	ns	ns	ns	ns	ns	ns	ns	ns	*	ns	ns

Treatments: control, FI_H_2_O; regulated deficit irrigation, RDI_H_2_O; nanosilicon exogenous additive, FI_SiNPs; combined regulated deficit irrigation and nanosilicon exogenous additive, RDI_SiNPs. The significance of three-way ANOVA for water (W), biostimulants (B), and genotype (G) is shown in each panel. ns, no significant difference; *, *p* <0.05; **, *p* < 0.01; ***, *p* < 0.001.

Compared to FI treatment, RDI treatment significantly delayed the silking stage in both H_2_O and SiNPs treatments. Compared to H_2_O treatment, SiNPs treatment significantly advanced the silking stage and shortened the anthesis-silking interval in both FI and RDI treatments. Additionally, the W × B interaction had a significant effect on the silking stage. Specifically, compared to H_2_O, SiNPs had no significant effect on the silking stage under FI treatment, while it advanced the silking stage under RDI treatment. There were significant differences in flowering stage, silking stage, and anthesis-silking interval among the three genotypes.

### 3.3 Effects of different irrigation methods and exogenous substances on WP, ET, biomass, and plant height

**Figure 1:**
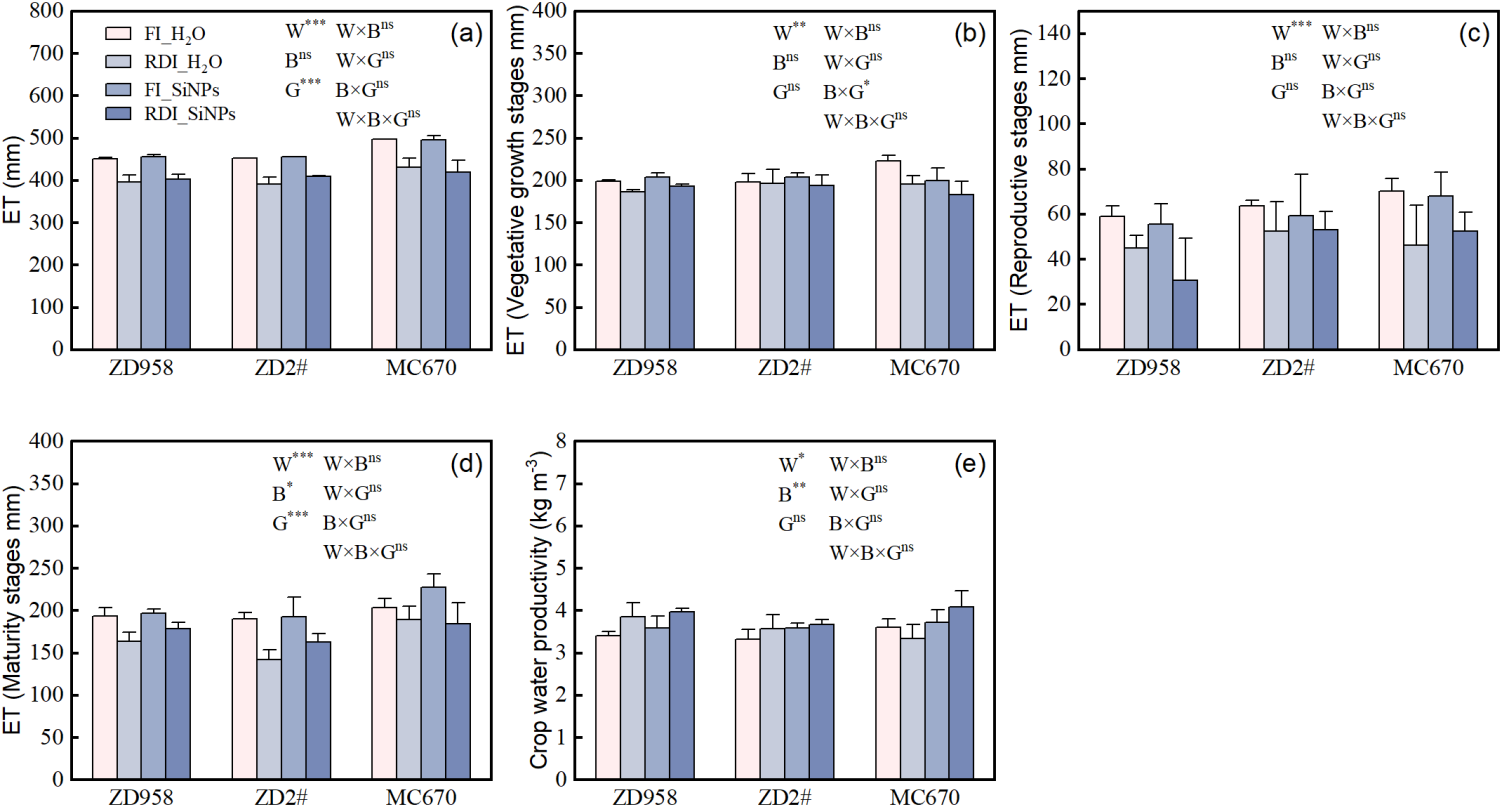
Effects of different water and exogenous substance treatments on evapotranspiration (ET) and water productivity (WP) of the three maize genotypes. **(a–d)** evapotranspiration (ET) and **(e)** crop water productivity (WP). Treatments: control, FI_H_2_O; regulated deficit irrigation, RDI_H_2_O; nanosilicon exogenous additive, FI_SiNPs; combined regulated deficit irrigation and nanosilicon exogenous additive, RDI_SiNPs. Values are means ± SD (n = 3 replications). The significance of three-way ANOVA for water (W), biostimulants (B), and genotype (G) is shown in each panel. ns, no significant difference; *, *p* < 0.05; **, *p* < 0.01; ***, *p* < 0.001.

Compared to FI treatment, RDI treatment significantly reduced ET during the entire growth period and at each growth stage in both H_2_O and SiNPs treatments (**Figures 1a-d**). Compared to H_2_O treatment, under both FI and RDI conditions, SiNPs treatment only had a significant effect on ET at the maturity stage. Genotype had significant effects on ET during both the entire growth period and the maturity stage. Overall, during the entire growth period, MC670 had the highest ET, followed by ZD2#, and ZD958 had the lowest. During the maturity stage, MC670 had the highest ET, followed by ZD958, and ZD2# had the lowest. Compared to FI treatment, RDI treatment significantly increased WP in both H_2_O and SiNPs treatments. Compared to H_2_O treatment, SiNPs treatment also significantly increased WP under both FI and RDI treatments (**Figure 1e**). WP was not significantly affected by genotype. Compared to FI_H_2_O (control treatment), WP increased by 13.45% under RDI_SiNPs treatment.

Compared to FI treatment, RDI treatment significantly reduced plant height and biomass during the entire growth period for all three genotypes under both H_2_O and SiNPs treatments (**Supplementary Figure S4**). Compared to H_2_O treatment, SiNPs treatment significantly increased plant height and biomass in all three genotypes under both FI and RDI treatments. The W × D interaction significantly affected plant height in all three genotypes, and the W × B interaction significantly influenced plant height in ZD2#. Additionally, the W × D interaction significantly affected biomass in ZD2#.

### 3.4 Effects of different irrigation methods and exogenous substances on LAI, leaf inclination angle, leaf area at different positions, and canopy net radiation interception

**Figure 2:**
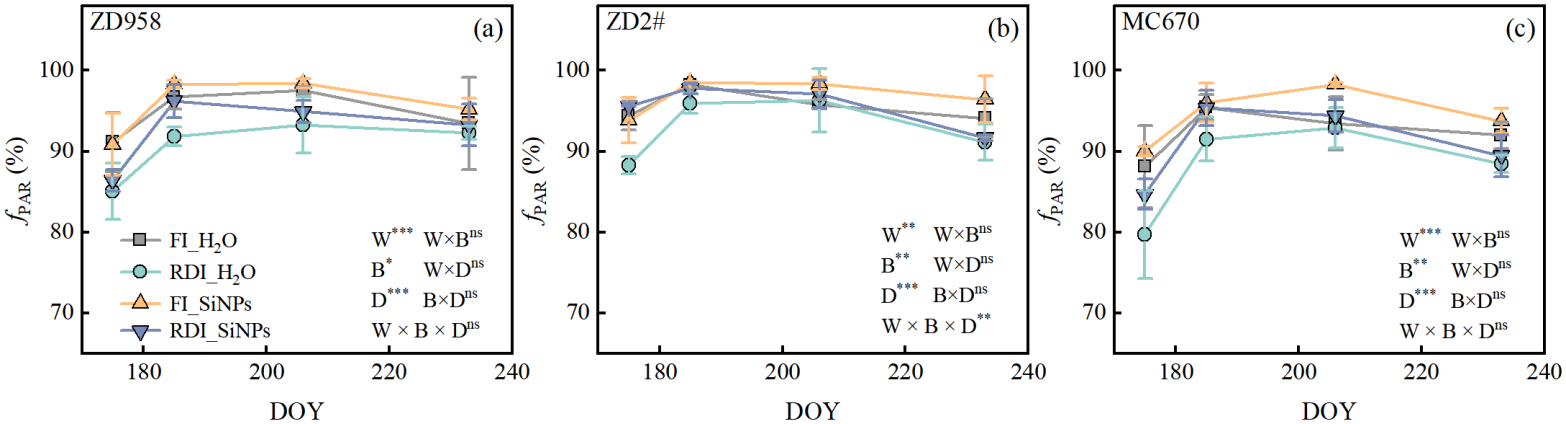
**(a–c)** Effects of different water and exogenous substance treatments on the fraction of photosynthetically active radiation (*f*_PAR_) of the three maize genotypes. Treatment: control, FI_H_2_O; regulated deficit irrigation, RDI_H_2_O; nanosilicon exogenous additive, FI_SiNPs; combined regulated deficit irrigation and nanosilicon exogenous additive, RDI_SiNPs. Values are means ± SD (n = 3 replications). The significance of three-way ANOVA for water (W), biostimulants (B), and date (D) is shown in each panel. ns, no significant difference; *, *p* <0.05; **, *p* < 0.01; ***, *p* < 0.001.

Compared to FI treatment, RDI treatment significantly reduced *f*_PAR_ in all three genotypes under both H_2_O and SiNPs treatments (**Figure 2**). Compared to H_2_O treatment, SiNPs treatment significantly increased *f*_PAR_ under both FI and RDI treatments.

**Figure 3:**
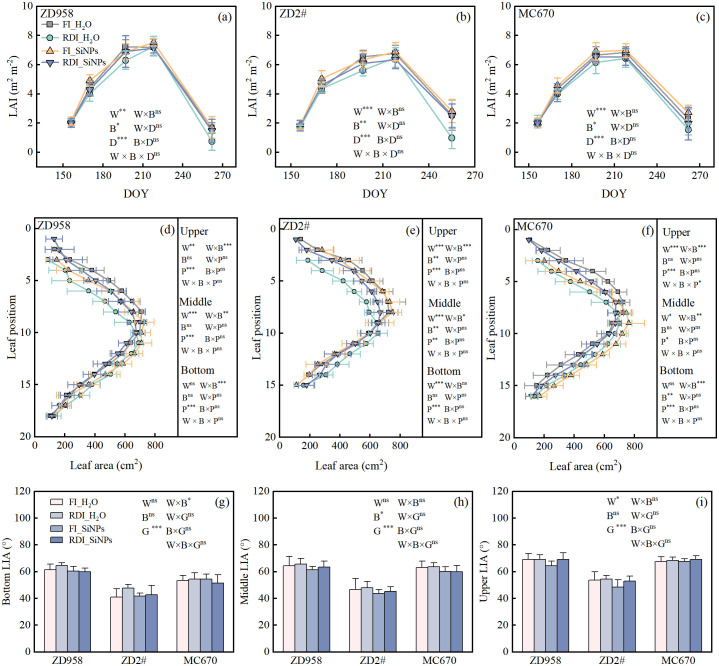
Effects of different water and exogenous substance treatments on leaf traits of the three maize genotypes. Treatments: control, FI_H_2_O; regulated deficit irrigation, RDI_H_2_O; nanosilicon exogenous additive, FI_SiNPs; combined regulated deficit irrigation and nanosilicon exogenous additive, RDI_SiNPs. **(a–c)** Leaf area index (LAI) (means ± SD, n = 3); **(d–f)** Leaf area at different plant positions (means ± SD, n = 6); **(g–i)** Leaf inclination angle (LIA) (means ± SD, n = 6). W represents the effect of water, B represents the effect of biostimulants, G represents the effect of genotypes, D represents the effect of date, and P represents the effect of position. ns, no significant difference; *, *p* <0.05; **, *p* < 0.01; ***, *p* < 0.001.

Compared to FI treatment, RDI treatment significantly reduced LAI in all three genotypes under both H_2_O and SiNPs treatments (**Figures 3a-c**). Compared to H_2_O treatment, SiNPs treatment significantly increased LAI in all three genotypes under both FI and RDI treatments. Significant differences in LAI were observed across the entire growth period among the three genotypes. Analysis of leaf position revealed that leaf area gradually increased with higher leaf positions in the lower part of the plant, reached a peak in the middle part, and then gradually decreased with higher leaf positions in the upper part of the plant (**Figures 3d-f**). In the upper part of the plant, RDI treatment significantly reduced LAI in both H_2_O and SiNPs treatments compared to FI treatment. Moreover, a significant W×B interaction was observed in the upper part. Specifically, compared to H_2_O treatment, SiNPs treatment reduced leaf area under FI conditions while increased leaf area under RDI conditions.

Irrigation methods significantly increased leaf inclination angle only in the upper part of the plant (**Figures 3g-i**). Compared to H_2_O treatment, SiNPs treatment significantly reduced the leaf inclination angle in the middle part of the plant at the ear position. Significant differences in leaf inclination angle were observed among genotypes in the upper, middle, and lower parts of the plant. A significant W × B interaction was observed in the lower part of the plant for leaf inclination angle.

### 3.5 Effects of different irrigation methods and exogenous substances on leaf gas exchange parameters

**Figure 4:**
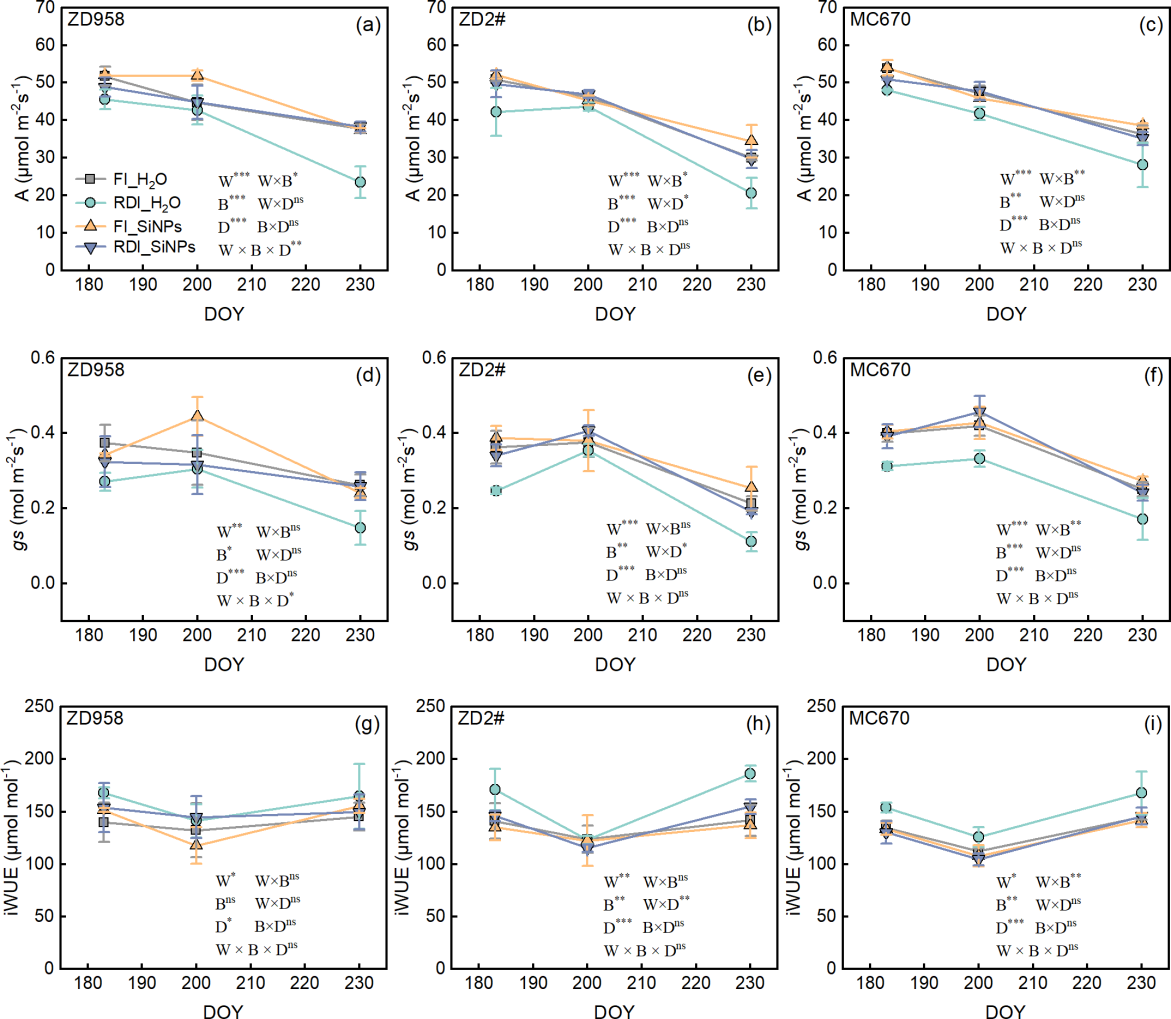
Effects of different water and exogenous substance treatments on leaf gas exchange and intrinsic water use efficiency of the three maize genotypes. **(a–c)** net photosynthesis rate (*A*), **(d–f)** stomatal conductance (*g*_s_), **(g–i)** intrinsic water use efficiency (iWUE). Treatments: control, FI_H_2_O; regulated deficit irrigation, RDI_H_2_O; nanosilicon exogenous additive, FI_SiNPs; combined regulated deficit irrigation and nanosilicon exogenous additive, RDI_SiNPs. Values are means ± SD (n = 3 replications). The significance of three-way ANOVA for water (W), biostimulants (B), and date (D) is shown in each panel. ns, no significant difference; *, *p* <0.05; **, *p* < 0.01; ***, *p* < 0.001.

Compared to FI treatment, RDI treatment significantly reduced the photosynthetic rate (*A*) and stomatal conductance (*g*_s_) during the entire growth period for all three genotypes under both H_2_O and SiNPs treatments, while significantly increasing intrinsic water use efficiency (iWUE) (**Figure 4**). Compared to H_2_O treatment, SiNPs treatment significantly increased *A* and *g*_s_ during the entire growth period for all three genotypes under both irrigation methods (FI and RDI), but decreased iWUE for ZD2# and MC670.

### 3.6 Effects of different irrigation methods and exogenous substances on SPAD and stomatal density

**Figure 5:**
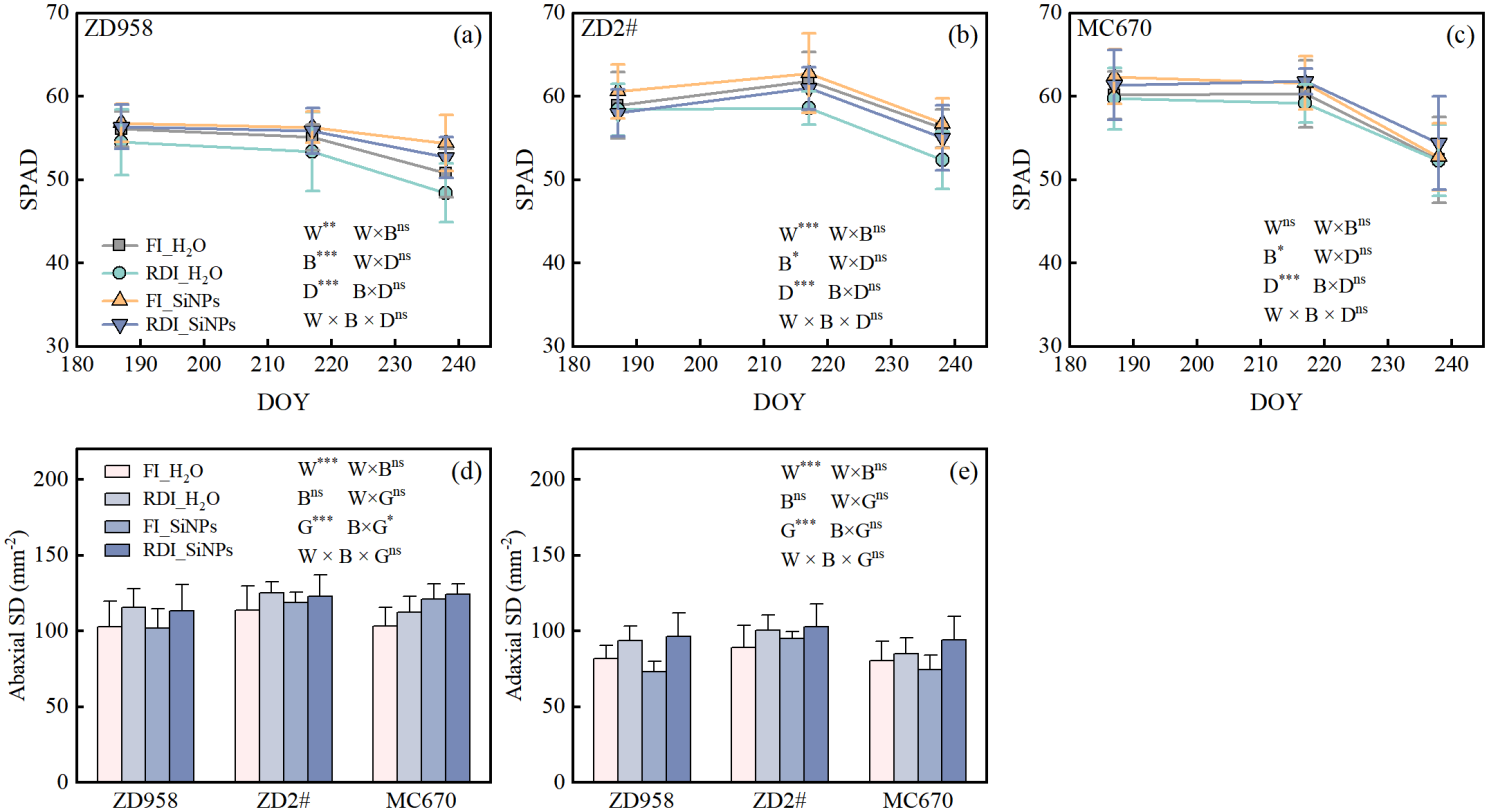
Effects of different water and exogenous substance treatments on SPAD and stomatal density. **(a–c)** temporal changes in SPAD values (means ± SD, n = 12); **(d, e)** stomatal density (*SD*) (means ± SD, n = 9). Treatments: control, FI_H_2_O; regulated deficit irrigation, RDI_H_2_O; nanosilicon exogenous additive, FI_SiNPs; combined regulated deficit irrigation and nanosilicon exogenous additive, RDI_SiNPs. W represents the effect of water, B represents the effect of biostimulants, G represents the effect of genotypes, and D represents the effect of date. ns, no significant difference; *, *p* < 0.05; **, *p* < 0.01; ***, *p* < 0.001.

Compared to FI treatment, RDI treatment significantly reduced SPAD during the entire growth period under H_2_O and SiNPs treatments for ZD958 and ZD2#, while the effect on MC670 was not significant (**Figures 5a-c**). Compared to H_2_O treatment, SiNPs treatment significantly increased SPAD during the entire growth period under both FI and RDI treatments for all three genotypes. Stomatal density (*SD*) on both adaxial and abaxial leaf surfaces was significantly affected by irrigation treatments (**Figures 5d, e**). But it was less influenced by exogenous substances. Compared to FI treatment, RDI treatment significantly increased adaxial and abaxial *SD* under both H_2_O and SiNPs treatments. The addition of exogenous substances had no significant effect on adaxial or abaxial *SD*. Significant differences were observed among genotypes under both adaxial and abaxial *SD*.

### 3.7 Physiological regulation mechanisms of yield and water productivity

**Figure 6:**
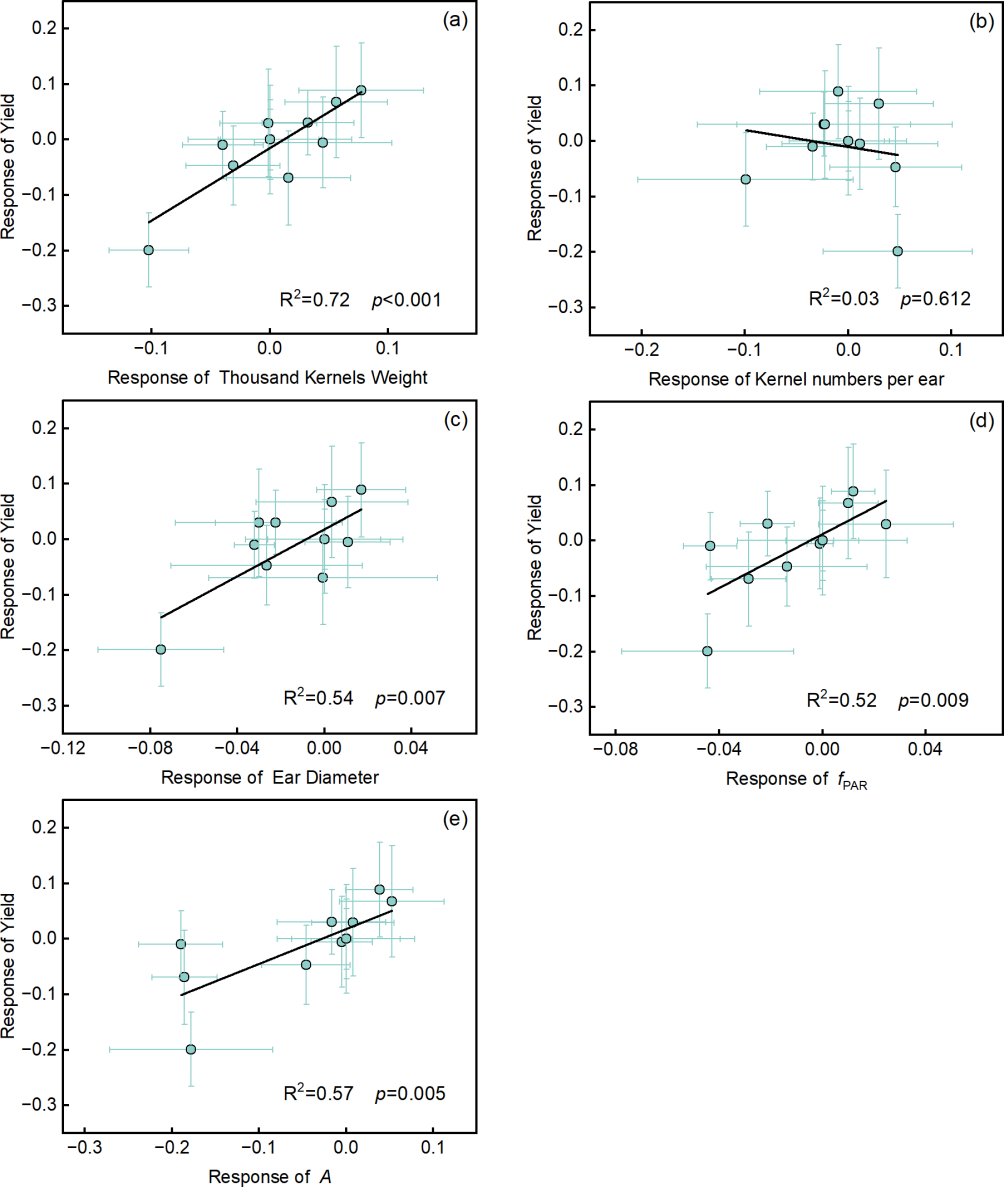
The relationship of yield with yield components, the canopy radiation interception (*f*_PAR_) and net photosynthetic rate (*A*). **(a)** thousand kernels weight, **(b)** kernels number per ear, **(c)** ear diameter, **(d)** the canopy radiation interception (*f*_PAR_), **(e)** net photosynthetic rate (*A*). Values are the response value of each indicator ± SD (n = 3 replications). Standard deviation was calculated using the error propagation law. The R² and *p* values are displayed in each panel.

To further explore the mechanisms by which deficit irrigation and SiNPs affect yield and water productivity (WP), we conducted a correlation analysis between yield and yield components, and physiological growth indicators (**Figure 6**). Yield showed a significant positive correlation with thousand-kernel weight (R² = 0.72, *p* < 0.001) and ear diameter (R² = 0.54, *p* < 0.01), but was not significantly correlated with the kernel numbers per ear (R² = 0.03, *p* = 0.612). Similarly, *f*_PAR_ (R² = 0.52, *p* < 0.01) and *A* (R² = 0.57, *p* < 0.01) were significantly positively correlated with yield.

**Figure 7:**
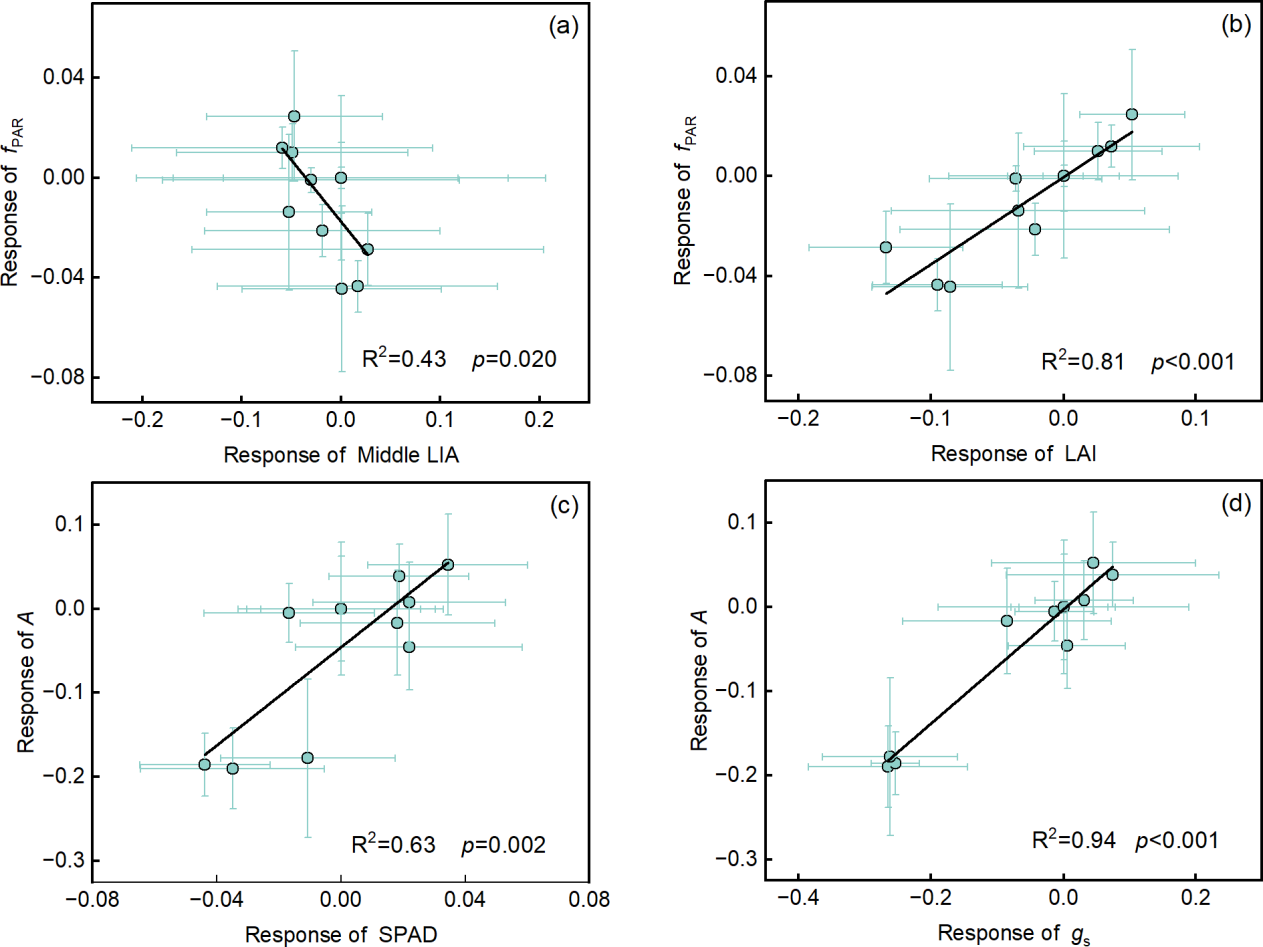
The relationship of the fraction of photosynthetically active radiation (*f*_PAR_) with leaf traits **(a-b)** and net photosynthesis rate with SPAD **(c)** and stomatal conductance (*g*_s_) **(d)**. Values are the response value of each indicator ± SD (n = 3 replications). Standard deviation was calculated using the error propagation law. The R² and *p* values are displayed in each panel.

A significant linear negative correlation is observed between middle layer leaf inclination angle (LIA) and *f*_PAR_ (R² = 0.43, *p* < 0.05) (**Figure 7a**), while bottom layer LIA and upper layer LIA was not significantly correlated with *f*_PAR_ (**Supplementary Figures S5a, b**). LAI (R² = 0.81, *p* < 0.001) (**Figure 7b**) and plant height (R² = 0.79, *p* < 0.001) (**Supplementary Figure S5c**) were highly positively correlated with *f*_PAR_. Both SPAD (R² = 0.63, *p* < 0.01) (**Figure 7c**) and *g*_s_ (R² = 0.94, *p* < 0.001) (**Figure 7d**) show a significant linear positive correlation with *A*.

**Figure 8:**
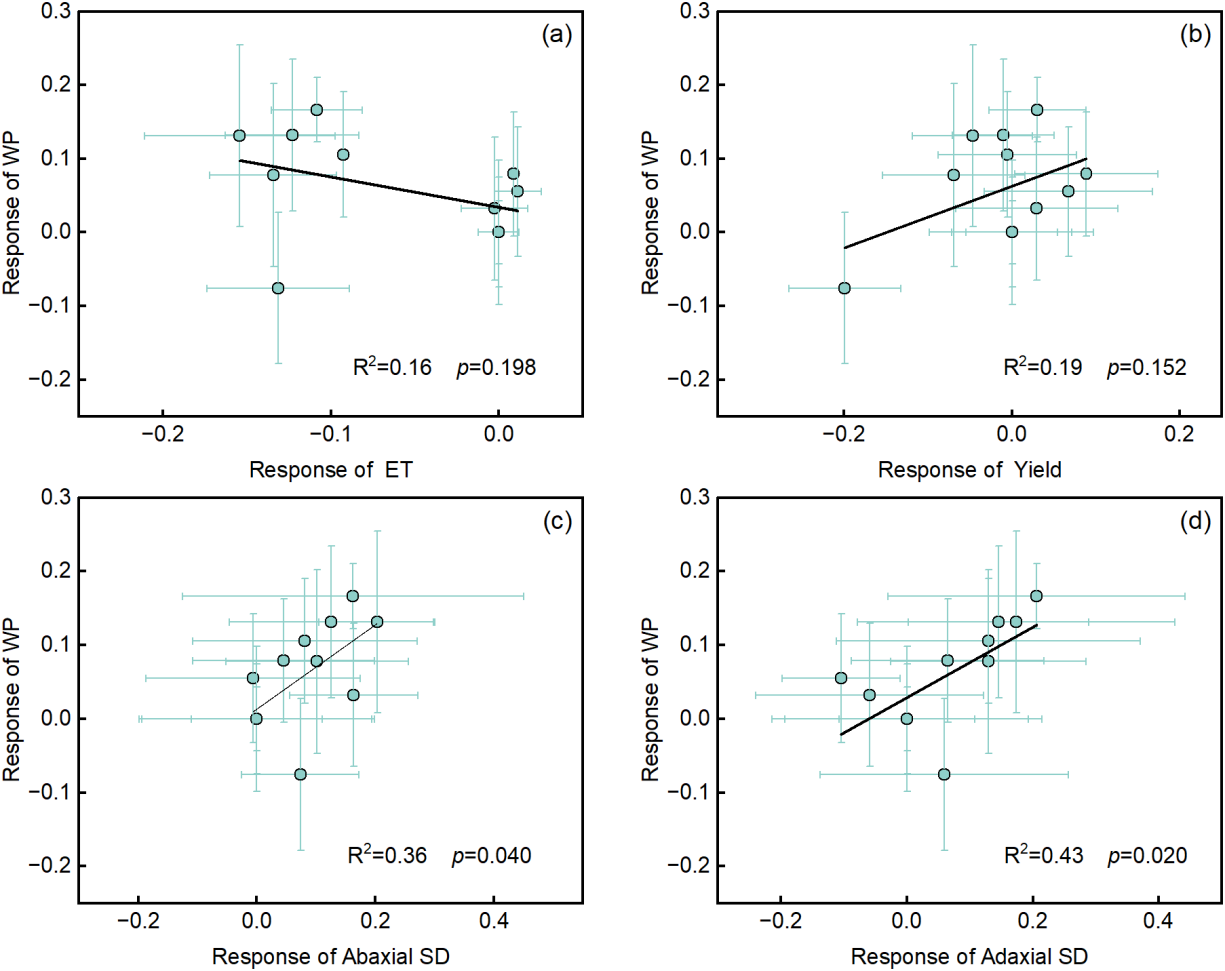
The relationship of crop water productivity (WP) with ET, yield, and stomatal density (*SD*). **(a)** evapotranspiration (ET), **(b)** yield, **(c)** abaxial *SD*, and **(d)** adaxial *SD*. Values are the response value of each indicator ± SD (n = 3 replications). Standard deviation was calculated using the error propagation law. The R² and *p* values are displayed in each panel.

We found that WP was not significantly correlated with ET and yield (**Figures 8a,b**). iWUE was not significantly correlated with WP (**Supplementary Figure S5d**); however, both abaxial stomatal density (*SD*) (R² = 0.36, *p* < 0.05) and adaxial *SD* (R² = 0.43, *p* < 0.05) showed significant linear positive correlations with WP (**Figures 8c, d**). Additionally, abaxial *SD* and adaxial *SD* were not significantly correlated with iWUE (**Supplementary Figures S5e , f**).

## 4 Discussion

**Figure 9:**
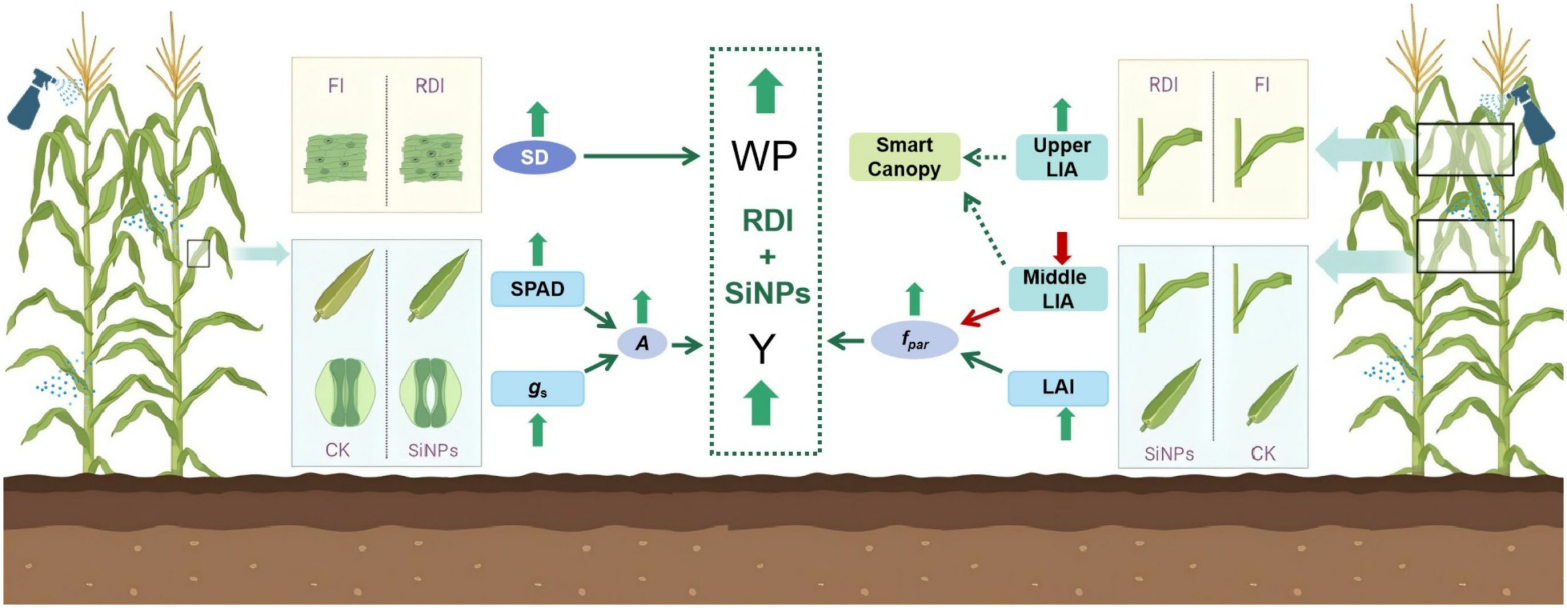
Mechanism of synergistic effects between RDI and SiNPs. FI and RDI represent full irrigation and regulated deficit irrigation, respectively. H_2_O and SiNPs represent water treatment for control and SiNPs treatment, *SD* represents stomatal density, SPAD represents soil plant analysis development, *g*_s_ represents stomatal conductance, *A* represents net photosynthesis rate, *f*_PAR_ represents the fraction of photosynthetically active radiation, LIA represents leaf inclination angle, LAI represents leaf area index, WP represents water productivity, Y represents yield.

This study systematically analyzed the effects and synergistic regulatory mechanisms of exogenous applications (H_2_O and SiNPs) and irrigation regimes (FI and RDI) on maize physiology, growth, yield, and water productivity (WP). Overall, while RDI caused a slight reduction in maize yield, it significantly improved WP by enhancing stomatal density (*SD*), achieving a favorable balance between yield compensation and water use efficiency. The application of SiNPs significantly increased maize yield and WP under both FI and RDI conditions. Additionally, it notably improved thousand-kernel weight, biomass, plant height, leaf area index (LAI), fraction of photosynthetically active radiation (*f*_PAR_), net photosynthetic rate (*A*), stomatal conductance (*g*_s_), and SPAD values. Moreover, it shortened the anthesis-silking interval (ASI) and significantly reduced the leaf inclination angle (LIA) of the middle canopy. Further analysis revealed that maize yield was significantly positively correlated with ear diameter, thousand kernel weight, *A*, and *f*_PAR_. The increase in yield mediated by SiNPs can be attributed to the following mechanisms: (1) enhancing *A* by increasing SPAD and *g*_s_; (2) improving *f*_PAR_ by reducing middle-layer LIA and increasing LAI, which together promoting a synergistic effect on boosts maize yield (**Figure 9**).

Drought stress limits water uptake in plants and inhibits their growth and development, ultimately negatively impacting crop yield. This study showed that SiNPs significantly increased maize yield (**Table 2**) and WP (**Figure 1**) under both FI and RDI conditions. Several studies have demonstrated that Si or SiNPs can increase crop yield under drought stress (Zhou et al., 2024; Alsaeedi et al., 2019) while achieving high water productivity (Ahmadian et al., 2021; Zulfiqar et al., 2024; Dou et al., 2023), which is consistent with the findings of this study. Compared to the FI_H_2_O treatment, yield of RDI_SiNPs treatment for ZD958, ZD2#, and MC670 has the variation of 3.05%, -0.52%, and -4.67%, respectively. However, compared to the RDI_H_2_O treatment, yield under the RDI_SiNPs treatment for ZD2# and MC670 significantly increased by 6.88% and 18.98%, respectively. This indicates that SiNPs application can effectively alleviate the negative effects of mild water deficit on yield, with the degree of impact varying among genotypes. Additionally, we found that mild RDI significantly increased maize *SD* (**Figure 5**), which is consistent with the results of Zhao et al. (2014). *SD* showed a significant positive correlation with WP (**Figures 8c, d**). This is because denser stomata can minimize the diffusion path of CO_2_ from stomata to mesophyll cells, thereby enhancing water use efficiency (Al-Salman et al., 2023). However, iWUE did not show a significant correlation with WP. This may be attributed to the fact that iWUE is typically measured at the leaf level, whereas WP serves as an indicator at the canopy or whole-plant level. When canopy structure affects light distribution and water utilization, the relationship between iWUE and WP may be diminished.

Under drought stress, the yield-increasing effects of SiNPs application are mainly attributed to the enhancement of *f*_PAR_ and *A*, with the contribution of *f*_PAR_ to yield increase being slightly higher than that of *A* (**Figures 6d, e**). The *f*_PAR_ is an important indicator for evaluating crop yield and biomass production (Tan et al., 2018), reflecting the ability of the vegetation canopy to absorb photosynthetically active radiation, which provides sufficient energy and material basis for crop growth and development, thereby directly promoting yield improvement (Raza et al., 2021; Zhang et al., 2024). Studies have shown that *f*_PAR_ is one of the most important variables in crop yield prediction (Clevers, 1997), which is consistent with the results of this study. The increase in net photosynthetic rate (*A*) indicates that plants can more efficiently assimilate CO_2_ and convert light energy into chemical energy (ATP and NADPH) (Long et al., 2015), supporting the synthesis and accumulation of photosynthetic products and yield.

The increase in *f*_PAR_ induced by SiNPs application was related to the increase in leaf area index (LAI) and the reduction in middle-layer leaf inclination angle (LIA), with the LAI having a greater impact on *f*_PAR_ than middle-layer LIA on that (**Figures 7a, b**). Liao et al. (2024) found plant leaf area is significantly reduced due to water deficit, leading to a decrease in canopy coverage and subsequently reducing the interception and absorption efficiency of *f*_PAR_, which is consistent with this study. Several related studies have observed that Si or SiNPs enhance leaf area expansion in various crops (Guo et al., 2006; Abd-El-Aty et al., 2024), improving light interception and accelerating plant growth (Alharbi et al., 2022). This study confirms the SiNPs’ role in expanding maize leaf area and optimizing resource allocation by regulating leaf area across different parts of the plant. The highly significant interaction between upper-layer leaf area (W × B) (**Figures 3d-f**) revealed that the impact of SiNPs on canopy structure was mainly derived from improvements in the upper canopy structure. Specifically, SiNPs promote leaf cell elongation under RDI condition, while it exhibited the ability to suppress excessive leaf growth in maize under full irrigation conditions.

Leaf angle influences photosynthesis, canopy structure, and crop yield. While upright maize architecture has been linked to yield improvement (Pendleton et al., 1968), recent studies propose optimizing leaf angle distribution across canopy layers. The “Smart Canopy” concept suggests upright leaves at the top, moderately upright in the middle, and more horizontal at the bottom (Tian et al., 2024; Ort et al., 2015). This study showed that RDI increased the leaf inclination angle (LIA) of upper-layer leaves, creating a more upright orientation, while SiNPs reduce the LIA of middle-layer leaves, flattening them. This aligns with the “Smart Canopy” model, preventing light saturation in the upper canopy and enhancing radiation interception by middle-layer leaves, which are key for maximizing solar radiation and improving light use efficiency (Fan et al., 2023). Liu et al. (2012) found that nano-silica reduced the leaf inclination angle in rice, consistent with our findings. The *LIGULELESS1* (LG1) gene regulates leaf angle by affecting leaf collar formation through auxin transport, highlighting auxin’s role in controlling maize leaf angle (Zhong et al., 2025). Li et al. (2024) found that SiNPs can influence auxin biosynthesis. We speculate that SiNPs may alter auxin synthesis, affecting leaf cell growth rate and inclination angle. However, the molecular mechanisms and pathways remain unclear and need further investigation. Additionally, SiNPs regulate *f*_PAR_ through LAI and middle-layer LIA, but the quantitative relationship between their synergistic effects on *f*_PAR_ has not been fully analyzed. Future research could integrate dynamic canopy models to explore their impact on light interception efficiency, uncover optimized mechanisms, and determine contribution rates, providing theoretical support for designing ideal crop architectures.

Under RDI, the application of SiNPs led to an increase in *A* primarily due to an increase in *g*_s_, followed by an increase in SPAD value (**Figures 7c, d**). Several studies have observed that Si or SiNPs can enhance *A*, *g*_s_, and SPAD under drought conditions (Maghsoudi et al., 2016; Bhardwaj and Kapoor, 2021; Nazim et al., 2024). Drought stress reduces *A* primarily by decreasing *g*_s_ due to lowered plant water potential (Xue et al., 2021). Si effectively alleviates drought stress by enhancing *g*_s_, thus mitigating the decline in photosynthesis (Liu et al., 2015). This improvements is closely associated with increased leaf water potential and relative water content (Liu et al., 2015). The potential mechanism may be that silicon forms a silicified protective layer on the leaf surface, which limits water loss and alleviates leaf dehydration (Yuvaraj et al., 2023). Chlorophyll, as a key molecule for light energy capture in photosynthesis, closely influences the electron transport efficiency of photosystem II (PSII) and photosystem I (PSI) (Komenda and Sobotka, 2019). Drought stress usually leads to structural damage in chloroplasts, including swelling, deformation, and even disintegration of thylakoid structures, thereby inhibiting both the light and dark reactions of photosynthesis (Zhang et al., 2015). However, the application of silicon or SiNPs can effectively mitigate stress-induced damage to chloroplasts (Feng et al., 2010; Younis et al., 2020). These benefits arise from the fact that silicon or SiNPs enhance the stability of organelle membranes, protect chloroplast membranes from disintegration, and maintain structural integrity (Xu et al., 2022). Some research suggests that SiNPs seed soaking treatment can catalyze the production of reactive oxygen species, which activates antioxidant signaling and the expression of downstream drought-responsive genes (Kang et al., 2025). This preventive mechanism can enhance crop drought resistance and improve yield (Kang et al., 2025).

## 5 Conclusion

The SiNPs enhance maize yield and crop water productivity (WP) through two pathways: first, by improving leaf photosynthetic rate and chlorophyll stability, thereby enhancing light energy conversion efficiency; second, by optimizing canopy structure and increasing the interception of photosynthetically active radiation. Meanwhile, RDI significantly increased WP by inducing higher stomatal density. The combined application of SiNPs and RDI in the arid northwest region resulted in yield maintenance (with variations within 5%) and a concurrent increase in WP of 11.11 – 17.62%. The innovation of this study is the elucidation of the synergistic mechanism of nano-silicon in “canopy light energy capture–dynamic stomatal regulation–enhanced carbon assimilation”. Additionally, a technical model combining mild RDI with seed soaking in 40 mg L^−1^ SiNPs and foliar spraying of 150 mg L^−1^ SiNPs was proposed, providing a reference strategy for efficient water resource utilization and stable yield regulation in maize production in arid regions.

## Data Availability

The raw data supporting the conclusions of this article will be made available by the authors, without undue reservation.
