# Supplementary figures and images for: Silicon nanoparticles enhance maize yield and water productivity via regulating photosynthesis and canopy structure under mild regulated deficit irrigation
Source: Front Plant Sci. 2026 Jan 6;16:1691443. doi: 10.3389/fpls.2025.1691443 (PMC12816220; doi:10.3389/fpls.2025.1691443)

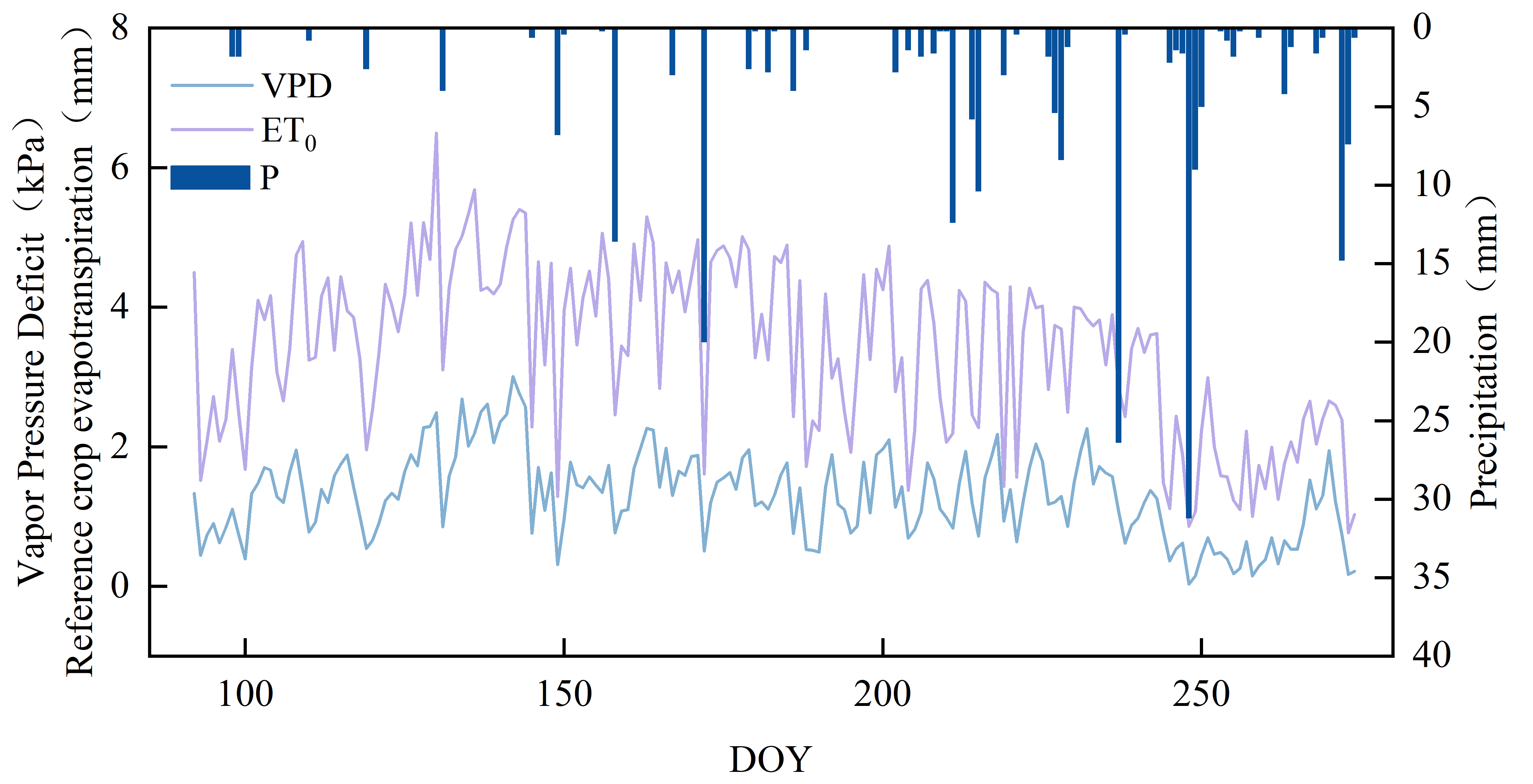

Supplement: Supplementary file 1 [file Image1.tif]

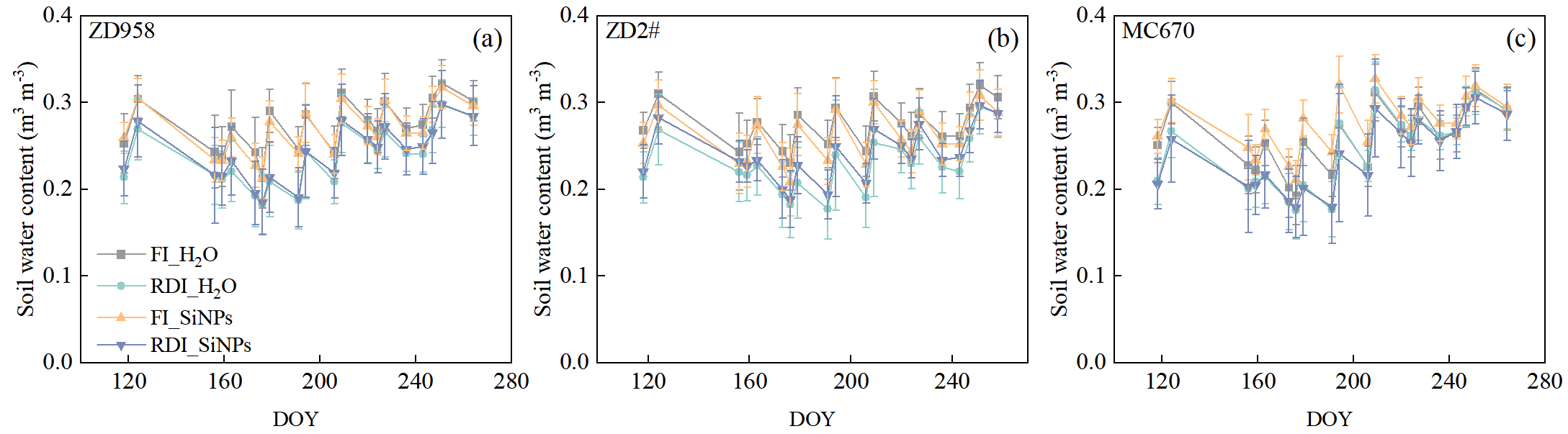

Supplement: Supplementary file 2 [file Image2.tif]

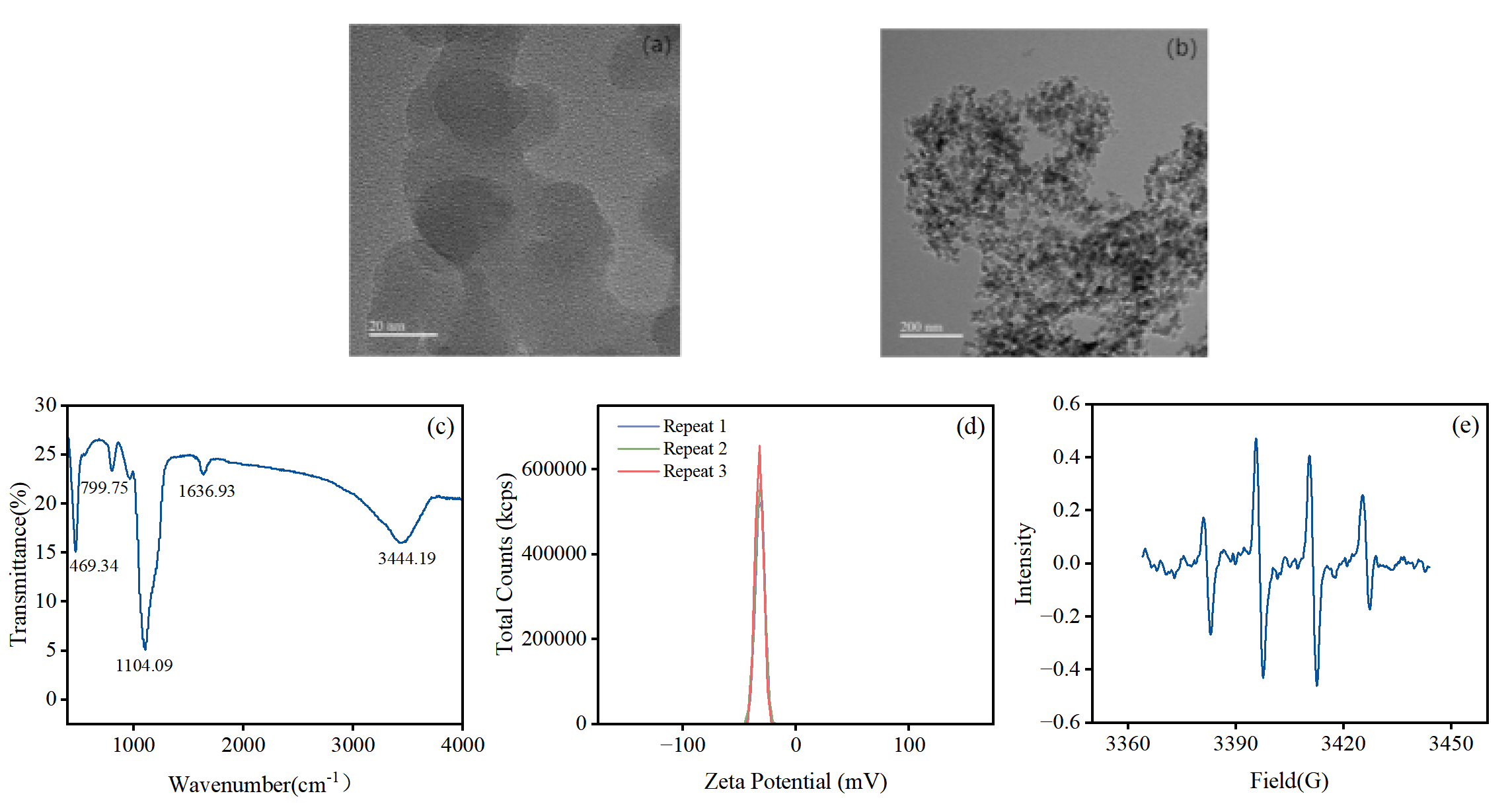

Supplement: Supplementary file 3 [file Image3.tif]

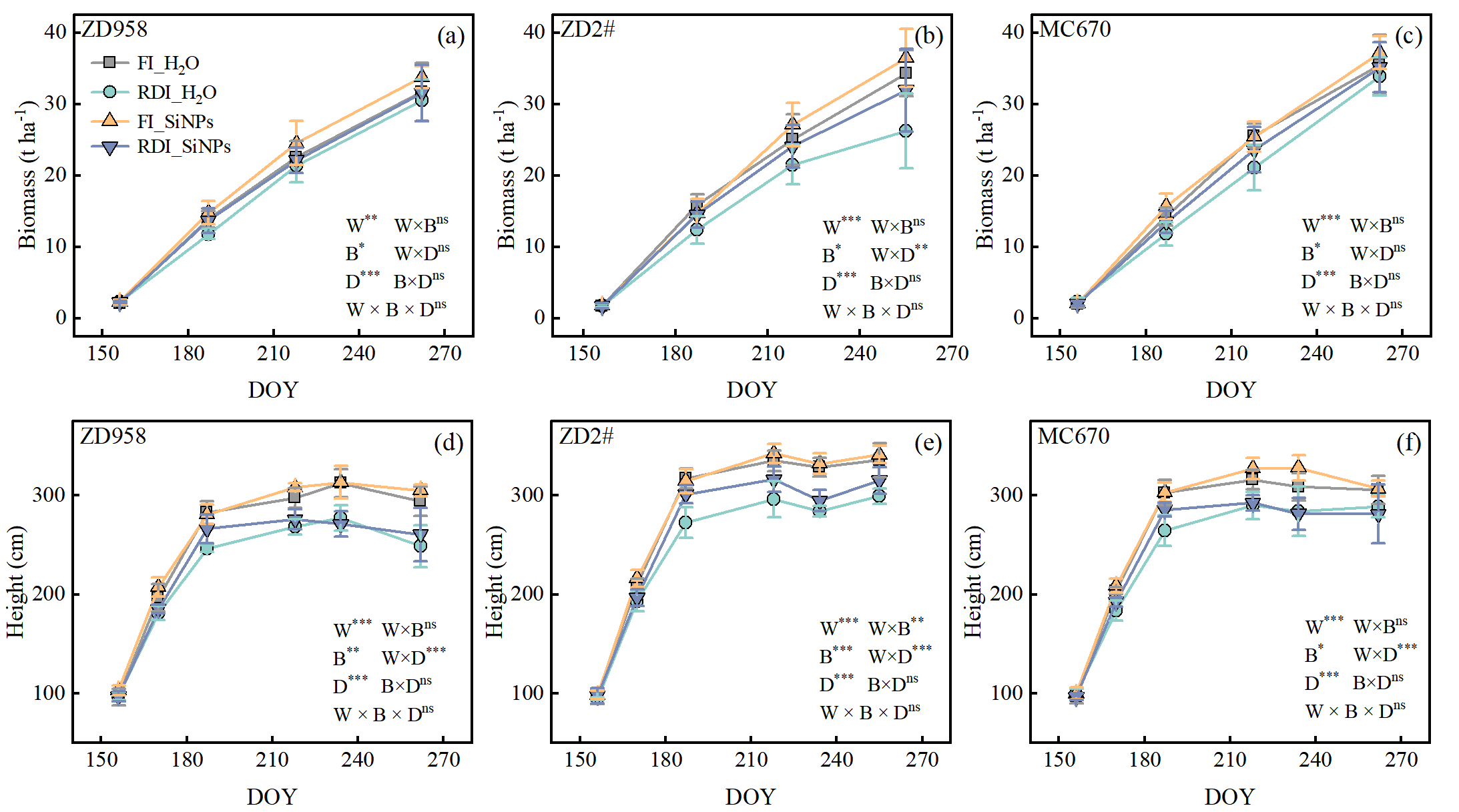

Supplement: Supplementary file 4 [file Image4.tif]

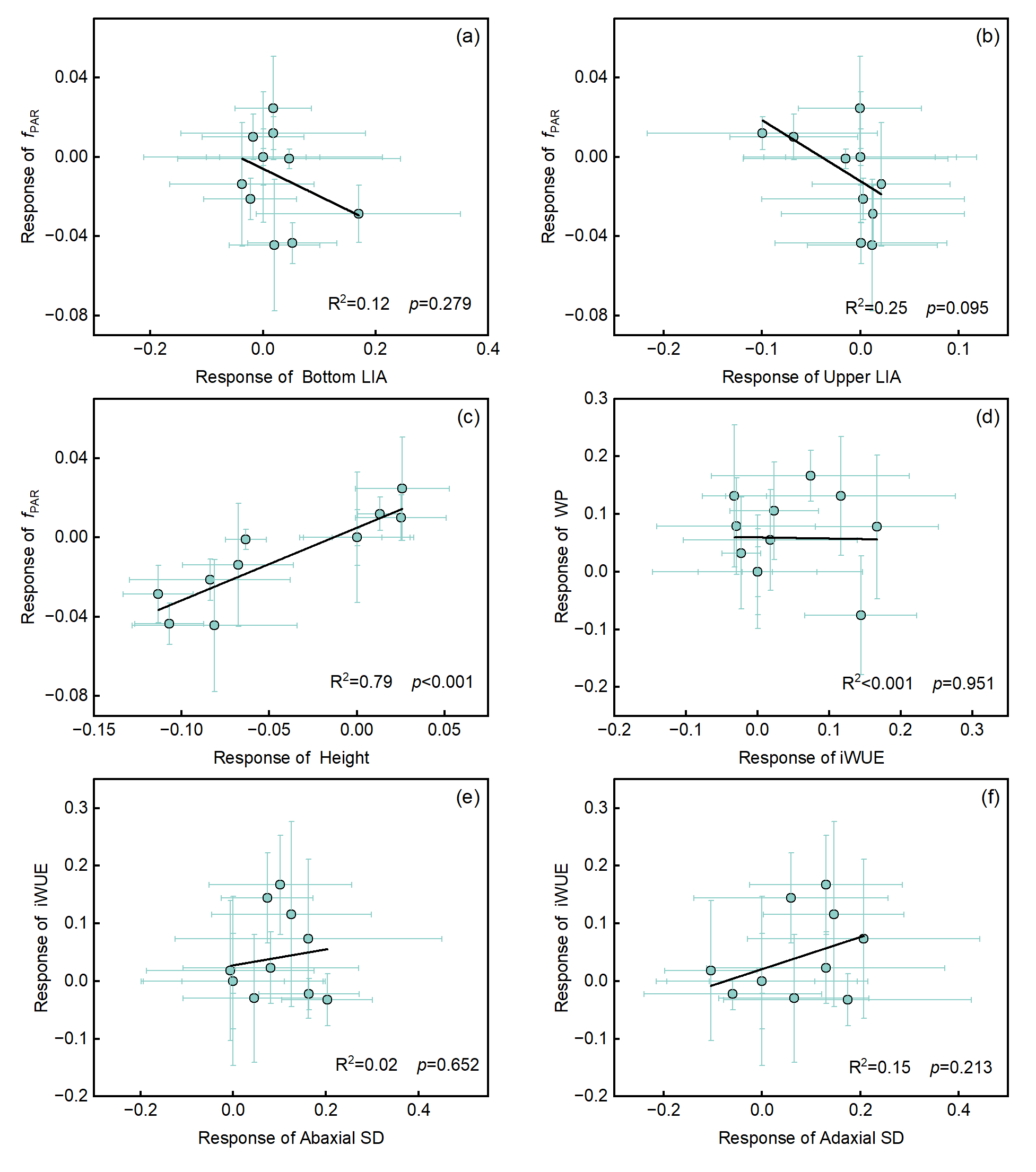

Supplement: Supplementary file 5 — Supporting information. [file Image5.tif]
